# In vitro Susceptibilities of Methicillin-Susceptible and Resistant *Staphylococci* to Traditional Antibiotics Compared to a Novel Fluoroquinolone

**DOI:** 10.1186/s12348-020-0200-0

**Published:** 2020-02-27

**Authors:** Kenneth C. Fan, James Lin, Nicolas A. Yannuzzi, Hasenin Al-Khersan, Nimesh A. Patel, Jorge Maestre-Mesa, Mustafa Zaidi, Darlene Miller, Harry W. Flynn

**Affiliations:** Department of Ophthalmology, Bascom Palmer Eye Institute, Miller School of Medicine, University of Miami, Miami, FL USA

**Keywords:** Antibiotic resistance, Endophthalmitis, Fluoroquinolone, Minimum inhibitory concentration, S*taphylococcus aureus*, *Staphylococcus epidermidis*, Vitreous

## Abstract

**Background:**

To assess the in-vitro efficacy of delafloxacin, a new fourth generation fluoroquinolone, against *Staphylococcus* vitreous isolates from patients with clinically diagnosed endophthalmitis. This is the first investigation of delafloxacin in ocular tissues.

**Methods:**

Intravitreal isolates of culture-proven *S. aureus* and *S. epidermidis* were identified between 2014 and 2018. Minimum inhibitor concentrations (MIC) were determined using ETEST strips. The antibiotic susceptibilities were tested against a panel of drugs including glycopeptides such as vancomycin, as well as traditional and newer fluoroquinolones (levofloxacin, moxifloxacin, and delafloxacin).

**Results:**

Of 45 total isolates identified between 2014 and 2018, 13% (6) were methicillin-resistant *S. aureus* (MRSA), 9% (4) were methicillin-sensitive *S. aureus* (MSSA), 53% (24) were methicillin-resistant *S. epidermidis* (MRSE), and 24% (11) were methicillin-sensitive *S. epidermidis* (MSSE). Among the fluoroquinolones, resistance rates were 61% for levofloxacin, 50% for moxifloxacin, and 12% for delafloxacin. Inter-class comparisons between delafloxacin and the two other fluoroquinolones demonstrated higher Gram-positive susceptibility to delafloxacin (*p* < 0.01). MIC90 values were lowest for delafloxacin (1.0 μg/mL) compared to levofloxacin (8.0 μg/mL) and moxifloxacin (8.0 μg/mL). Vancomycin was 100% effective against all isolates with MIC90 value of 0.75 μg/mL.

**Conclusion:**

Compared to levofloxacin and moxifloxacin, the newer fluoroquinolone delafloxacin demonstrated the lowest MICs values and lowest rates of resistance for Gram-positive in-vitro *S. epidermidis* and *S. aureus* vitreous isolates.

## Introduction

Endophthalmitis is a devastating intraocular condition caused by a variety of different microbiological organisms. The most common category of exogenous endophthalmitis is acute-onset post-operative, comprising 40–80% of all causes of endophthalmitis [1]. Other less common etiologies include post-injection and post-traumatic endophthalmitis [1, 2]. Across these mechanisms of infection, the most common causative organism is coagulase-negative *Staphylococcus* (CoNS) [1–6]. Prior studies have established high rates of antibiotic resistance in Gram-positive endophthalmitis vitreous isolates, with resistance to fluoroquinolones including ciprofloxacin and levofloxacin as high as 41% and 56%, respectively [5, 7, 8]. Furthermore, trends towards increasing drug resistance of Gram-positive vitreous isolates have been demonstrated [5, 8–11]. In light of these data, the development and investigation of newer, potentially more effective antibiotics for endophthalmitis is clinically important.

Delafloxacin is a new broad-spectrum fluoroquinolone that was approved by the FDA in June 2017 for oral and intravenous use in the treatment of acute bacterial skin and skin structure infections (ABSSSIs) after demonstrating non-inferiority to vancomycin and aztreonam [12]. To date, there are no reports demonstrating the effect of delafloxacin in intraocular tissue infections either in vivo or in vitro. The purpose of the current study is to investigate the possible role that delafloxacin may play in treating infectious endophthalmitis.

## Methods

Institutional review board approval was obtained from the University of Miami Miller School of Medicine Sciences Subcommittee for the Protection of Human Subjects and the research followed the tenets of the Declaration of Helsinski (IRB Protocol Study ID #20120897). The Ocular Microbiology Department database was searched to identify non-consecutive positive intravitreal isolates of culture-proven *Staphylococcus aureus* and S*taphylococcus epidermidis* organisms between January 1, 2014 and December 31, 2018. Records from the microbiology department were reviewed to confirm isolates and identify antimicrobial susceptibilities.

Isolates were cultured using standard microbiological procedures. Vitreous cultures were obtained at the time of vitreous tap or vitrectomy in patients with endophthalmitis. For vitreous tap samples, fluid was directly cultured onto 5% sheep blood and chocolate agar culture media. For vitrectomy samples, 30–50 mL of vitreous washings were filtered using a 0.45-μm filter, which were divided into segments and plated onto culture media, including 5% sheep blood and chocolate agar. Blood and chocolate agar plates underwent incubation at 35 °C for up to 2 weeks. Additional culture media, including thioglycollate broth, was submitted at the discretion of the ophthalmologist performing the culture.

**Fig. 1:**
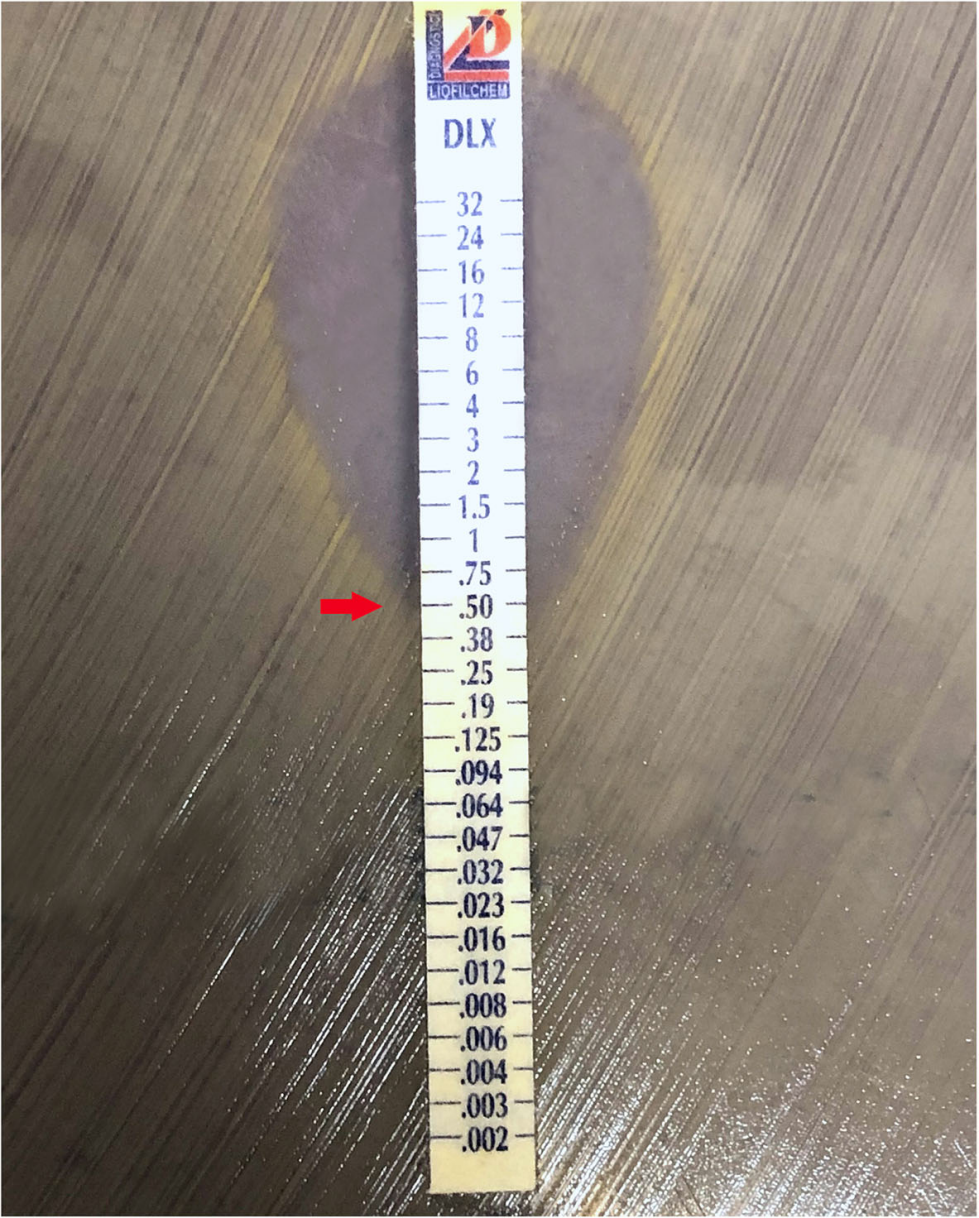
Growth of Gram-positive organisms from intravitreal endophthalmitis isolates on Mueller-Hinton agar with ETEST strip testing for antibiotic susceptibilities and minimum inhibitory concentrations. The drug is eluted in immediate proximity to the plastic carrier, creating a gradient of drug concentrations to measure the minimum concentration required for the inhibition of growth. This figure represents inhibition of growth (red arrow), a minimum concentration of 0.5 μg/ml.

A standard inoculum (1 × 10^8^ CFU/mL) for each isolate was placed on Mueller-Hinton agar. Minimum inhibitor concentrations (MIC) were determined using ETEST strips (bioMérieux, Marcy l’Etoile, France) placed according to manufacturer’s instructions, and susceptibilities were based on breakpoints from Clinical & Laboratory Standards Institute (CLSI) guidelines (see Fig. 1). MIC90 was calculated based on the concentration at which growth of all organisms were inhibited. Plates were incubated in a non-CO_2_ incubator and read after 18–24 h. The antibiotic susceptibilities were tested against vancomycin as well as traditional and newer fluoroquinolones (levofloxacin, moxifloxacin, and delafloxacin).

Organisms were graded as either susceptible or resistant to each antibiotic. Cases of indeterminate resistance were classified as antibiotic resistant. Pearson chi-squared testing was used to compare relative antibiotic susceptibility among each of the fluoroquinolone antibiotics. A *p* value < 0.05 was considered statistically significant. Statistical analysis was carried out using Stata 15 (StataCorp, College Station, TX).

## Results

Of 45 total isolates identified between 2014 and 2018, 13% (6) were methicillin-resistant *S. aureus* (MRSA), 9% (4) were methicillin-sensitive *S. aureus* (MSSA), 53% (24) were methicillin-resistant *S. epidermidis* (MRSE), and 24% (11) were methicillin-sensitive *S. epidermidis* (MSSE, see Table 1). Among the fluoroquinolones, resistance rates across all organisms were 60% for levofloxacin, 50% for moxifloxacin, and 12% for delafloxacin (see Table 1). Direct comparisons between delafloxacin and moxifloxacin across all *Staphylococcus* isolates demonstrated higher susceptibilities for delafloxacin (*p* < 0.01). Likewise, direct comparisons between delafloxacin and levofloxacin across all *Staphylococcus* isolates demonstrated higher susceptibilities for delafloxacin (*p* < 0.01). Vancomycin was effective against all isolates. For MSSA, vitreous isolate samples demonstrated a 50% rate of resistance for levofloxacin, 50% for moxifloxacin, and 0% for delafloxacin. MSSE displayed a similar trend, with 60% resistance for levofloxacin, 55% for moxifloxacin, and 27% for delafloxacin. For isolates that were resistant to methicillin (MRSA and MRSE), resistance rates increased significantly to 83%, 83%, and 33% rates of resistance against levofloxacin, moxifloxacin, and delafloxacin respectively, and for MRSE, 54%, 42%, and 25% rates of resistance against levofloxacin, moxifloxacin, and delafloxacin respectively (see Table 1).

**Table 1:** Antimicrobial activities of delafloxacin, levofloxacin, and moxifloxacin tested against intravitreal *Staphylococcus* isolates

Organism	Rates of resistance									
	All isolates	MIC90^a^	MSSA^b^	MIC90	MSSE^c^	MIC90	MRSA^d^	MIC90	MRSE^e^	MIC90
Levofloxacin	60% (26/43)	8.0	50% (2/4)	--	60% (6/10)	8.0	83% (5/6)	--	54% (13/24)	8.0
Moxifloxacin	50% (22/44)	8.0	50% (2/4)	--	55% (6/11)	8.0	83% (5/6)	--	54% (13/24)	8.0
Delafloxacin	12% (12/43)	1.0	0% (0/4)	--	27% (3/11)	0.8	33% (2/6)	--	25% (6/24)	0.8

^a^MIC90, minimum inhibitory concentration at which growth of 90% of organisms is inhibited, expressed in micrograms per milliliter (μg/ml), which were only calculated for microbes with more than 10 isolates

^b^MSSA, methicillin-sensitive *S. aureus*

^c^MRSA,methicillin-resistant *S. aureus*

^d^MSSE, methicillin-sensitive *S. epidermidis*

^e^MRSE, methicillin-resistant *S. epidermidis*

MIC90 values were lowest for delafloxacin (1.0 μg/mL) compared to levofloxacin (8.0 μg/mL) and moxifloxacin (8.0 μg/mL, see Table 1). The MIC90 for vancomycin was 0.75 μg/mL.

## Discussion

The most common microbes causing exogenous endophthalmitis are Gram-positive cocci, namely CoNS as well as *S. aureus* [1, 2, 5, 13]. In the Endophthalmitis Vitrectomy Study as well as some more recent studies, CoNS and *S. aureus* have remained at similar post-operative prevalence rates following cataract surgery through the decades (approximately 60% and 10%, respectively) [6, 10, 13]. Occurring at a rate of 0.056%, endophthalmitis after intravitreal injection is the second most common category of endophthalmitis, with CoNS comprising 38% of cases, followed by *Streptococcus* species and S*. aureus* [2].

Current empiric treatment of suspected bacterial endophthalmitis typically begins with intravitreal vancomycin and ceftazidime [1, 6, 14–16]. Regarding fluoroquinolones, intracameral and systemic treatment has gained interest in light of the favorable safety profile and low MIC levels of some agents like moxifloxacin [15, 17]. However, steadily increasing rates of antibiotic resistance to fluoroquinolones among CoNS have been reported (56% nonsusceptibility for ciprofloxacin and levofloxacin, 57% for moxifloxacin) [7], with these resistant microbes conferring poor visual outcomes [18].

The current study examines the in-vitro efficacy of a new fourth-generation fluoroquinolone, delafloxacin, for *S. epidermidis* and *S. aureus* endophthalmitis vitreous isolates in a single, tertiary care institution over a 4-year period. Delafloxacin is a promising new antibiotic that targets bacterial topoisomerase IV and DNA gyrase and demonstrates activity against Gram-positive bacteria including MRSA and Gram-negative bacteria such as *Pseudomonas aeruginosa (P. aeruginosa)* [15]. In vivo pharmacokinetic and pharmacodynamic studies have demonstrated rapid oral absorption of delafloxacin, high bioavailability (60–70%), and renal excretion [19]. Delafloxacin is a concentration-dependent fluoroquinolone, exhibiting in vivo MICs of ≤ 1 mg/L against *P. aeruginosa* and 0.25 mg/L for *Klebsiella pneumoniae*, which predicts efficacy against 75% and 78% of strains respectively [19–21]. In vitro studies have shown MIC levels for delafloxacin to be 3–5 times lower than other fluoroquinolones against Gram-positive microbes [21]. Additionally, multiple groups have shown its higher in vitro efficacy against Gram-positive organisms compared to other in-class agents [22, 23].

The findings in the current study demonstrate that delafloxacin is a more effective fluoroquinolone than either levofloxacin or moxifloxacin for in-vitro Gram-positive vitreous isolates, with lower nonsusceptibility rates for both *S. epidermidis* and *S. aureus.* This has been demonstrated in previous studies for ABSSSIs as well and now is confirmed for ocular tissues [23]. Additionally, for methicillin-resistant strains of *S. epidermidis* and *S. aureus*, rates of resistance were also comparatively lower for delafloxacin. MIC90 levels reported were significantly lower for delafloxacin (1.0 μg/mL) compared to 8.0 μg/mL for both levofloxacin and moxifloxacin, indicating that lower concentrations are required to reach therapeutic levels against vitreous isolates in vitro. This lower MIC represents an 8-fold decrease in concentration required for inhibition of growth compared to other fluoroquinolones, which had been previously reported as 3- to 5-fold lower in vitro [21].

Similar to prior studies, vancomycin was effective against all 45 isolates in this study, and it remains one of the most effective empiric treatments for endophthalmitis [5, 6, 9, 10, 24]. However, delafloxacin has the benefit of wider microbial coverage including Gram-negative organisms like *P. aeruginosa*, anaerobes, and atypical infections [25, 26]. Systemically, safety studies have not shown any QTc prolongation or evidence of phototoxicity often seen in other fluoroquinolones; however, some risk of tendonitis still exists [21]. Further investigations on the bioavailability, intraocular toxicity, and penetration of ocular tissues are required.

The limitations of this study include the analysis of only CoNS and *S. aureus* organisms, the retrospective design of the study, and lack of an intraocular formulary for delafloxacin. Expanding the analysis to both Gram-positive and Gram-negative organisms may lend further utility in determining the role of delafloxacin in endophthalmitis. Due to a retrospective design, stratification by post-operative, post-injection, post-traumatic, or other causes was not possible. ETEST strips were used for determination of MIC, which allowed only for close but still accurate approximation. Lastly, delafloxacin has only been approved in oral and intravenous formulations, and safety and pharmacokinetics studies have not been performed in intraocular tissues.

## Conclusion

Delafloxacin is an exciting new drug that demonstrates potential utility in endophthalmitis for *Staphylococcus* vitreous isolates due to low MIC levels and low comparative rates of nonsusceptibility even for methicillin-resistant organisms. Compared to levofloxacin and moxifloxacin, the newer fluoroquinolone delafloxacin demonstrated the lowest MICs values and lowest rates of resistance for Gram-positive in-vitro S. epidermidis and S. aureus vitreous isolates. The current landscape of rising antibiotic resistance makes the investigation of newer, more efficacious therapies important for improving clinical outcomes. Delafloxacin is an expensive drug, however, costing approximately $675 for a 5-day course of systemic treatment (compared to $51 for moxifloxacin) [15]. Therefore, future investigations on cost utility of the drug as well as wider-scale prospective studies will help define its promising role in the treatment of endophthalmitis.

## Data Availability

The datasets used and analyzed during the current study are available from the corresponding author upon reasonable request.

## Abbreviations

ABSSSI: Acute bacterial skin and skin structure infections
CLSI: Clinical & Laboratory Standards Institute
CoNS: Coagulase-negative Staphylococcus
MIC: Minimum inhibitory concentration
MRSA: Methicillin-resistant *Staphylococcus aureus*
MRSE: Methicillin-resistant *Staphylococcus epidermidis*
*MSSA*: Methicillin-sensitive *Staphylococcus aureus*
MSSE: Methicillin-sensitive *Staphylococcus epidermidis*
